# Development of a core outcome set for clinical trials in facial aging: study protocol for a systematic review of the literature and identification of a core outcome set using a Delphi survey

**DOI:** 10.1186/s13063-017-2104-3

**Published:** 2017-08-01

**Authors:** Daniel I. Schlessinger, Sanjana Iyengar, Arianna F. Yanes, Jill K. Henley, Hovik J. Ashchyan, Anastasia O. Kurta, Payal M. Patel, Umar A. Sheikh, Matthew J. Franklin, Courtney C. Hanna, Brian R. Chen, Sarah G. Chiren, Jochen Schmitt, Stefanie Deckert, Karina C. Furlan, Emily Poon, Ian A. Maher, Todd V. Cartee, Joseph F. Sobanko, Murad Alam

**Affiliations:** 1 Department of Dermatology, Feinberg School of Medicine, Northwestern University, 676 North St. Clair Street, Suite 1600, Chicago, IL USA; 2 Department of Dermatology, University of Pennsylvania, Philadelphia, PA USA; 3 Department of Dermatology, St. Louis University School of Medicine, St. Louis, MO USA; 4 Department of Dermatology, Penn State Hershey Dermatology, Hershey, PA USA; 5 Centre for Evidence-Based Healthcare, Medizinische Fakultät Carl Gustav Carus, TU Dresden, Dresden, Germany; 6 Division of Dermatologic Surgery, University of Pennsylvania, Philadelphia, PA USA; 7 Department of Otolaryngology, Feinberg School of Medicine, Northwestern University, Chicago, IL USA; 8 Department of Surgery, Feinberg School of Medicine, Northwestern University, Chicago, IL USA

**Keywords:** Core outcome set, Delphi, Consensus, Stakeholders, Aging, Systematic review

## Abstract

**Background:**

Facial aging is a concern for many patients. Wrinkles, loss of volume, and discoloration are common physical manifestations of aging skin. Genetic heritage, prior ultraviolet light exposure, and Fitzpatrick skin type may be associated with the rate and type of facial aging. Although many clinical trials assess the correlates of skin aging, there is heterogeneity in the outcomes assessed, which limits the quality of evaluation and comparison of treatment modalities. To address the inconsistency in outcomes, in this project we will develop a core set of outcomes that are to be evaluated in all clinical trials relevant to facial aging.

**Methods/design:**

A long list of measureable outcomes will be created from four sources: (1) systematic medical literature review, (2) patient interviews, (3) other published sources, and (4) stakeholder involvement. Two rounds of Delphi processes with homogeneous groups of physicians and patients will be performed to prioritize and condense the list. At a consensus meeting attended by physicians, patients, and stakeholders, outcomes will be further condensed on the basis of participant scores. By the end of the meeting, members will vote and decide on a final recommended set of core outcomes. Subsequent to this, specific measures will be selected or created to assess these outcomes.

**Discussion:**

The aim of this study is to develop a core outcome set and relevant measures for clinical trials relevant to facial aging. We hope to improve the reliability and consistency of outcome reporting of skin aging, thereby enabling improved evaluation of treatment efficacy and patient satisfaction.

**Trial registration:**

Core Outcome Measures in Effectiveness Trials (COMET) Initiative, accessible at http://www.comet-initiative.org/studies/details/737. Core Outcomes Set Initiative, (CSG-COUSIN) accessible at https://www.uniklinikum-dresden.de/de/das-klinikum/universitaetscentren/zegv/cousin/meet-the-teams/project-groups/core-outcome-set-for-the-appearance-of-facial-aging. Protocol version date is 28 July 2016.

**Electronic supplementary material:**

The online version of this article (doi:10.1186/s13063-017-2104-3) contains supplementary material, which is available to authorized users.

## Background

Aging of the facial skin is a natural process resulting from a complex combination of both intrinsic and extrinsic factors, such as genetic influences and exposure to sunlight [1]. Clinical features of skin aging include wrinkles, soft tissue/volume loss, mottled discoloration, loss of elasticity, dullness, and roughness. Histologically, aging of the skin is characterized by epidermal thinning, keratinocyte atypia, dermal elastosis, and loss of fibrillin- and collagen-containing structures in the dermis [1–3]. Because physical attractiveness is strongly correlated with self-esteem and quality of life, aging of the skin is an important concern encountered in dermatology [4–6].

In 2014, 10 million surgical and nonsurgical cosmetic procedures were performed in the United States alone [7]. Numerous interventions for the improvement of facial aging and appearance are available. Common treatments include, but are not limited to, injectable neurotoxins and fillers, fat reduction techniques, surgical facelifts, laser resurfacing, laser treatment of dyspigmentation and erythema, and skin tightening therapeutic radiofrequency and ultrasound treatments [2, 7]. According to ClinicalTrials.gov, more than 1000 trials relevant to facial skin aging are either in progress, actively recruiting, or completed [8].

However, there are few validated techniques for the evaluation of facial aging and appearance. Due to its subjective nature, facial aging is often assessed by newly created scales and devices, which may be unique to a particular study. Hence, it is difficult to adequately compare study results across trials. Similarly, comparisons of different therapies are also complicated by the inadequacy of outcome measures.

Selective outcome reporting bias, defined as results-based selection of outcomes for publication, is a concern in many clinical trials and affects many systematic reviews [9]. Specific organizations have been formed to counter this problem. The Core Outcome Measures in Effectiveness Trials (COMET) Initiative brings together researchers interested in developing a standardized set of core outcomes in various health-related fields [10]. A core outcome set (COS) is defined as an agreed minimum set of outcomes that is recommended to be measured and reported in all clinical trials.

Another organization, the Cochrane Skin Group - Core Outcome Set Initiative (CSG-COUSIN), is designed specifically to address COSs in dermatology by examining outcome measures in current research [11, 12]. CSG-COUSIN builds on the roadmap developed by the Harmonising Outcome Measures for Eczema (HOME) initiative for the process of COS development and implementation [13].

Currently, there is no COS established for facial aging. Through this study, we hope to create a standardized set of outcomes. This study has been registered with both the COMET and CSG-COUSIN organizations.

### Objective

The aim of this study is to develop an international COS relevant to clinical trials of facial aging. Through the use of a systematic literature review, stakeholder involvement, and consensus process, we hope to determine a short list of important outcomes that should be assessed in all related clinical trials, as well as the measures that may afford the best assessment of these outcomes.

## Methods/design

**Fig. 1:**
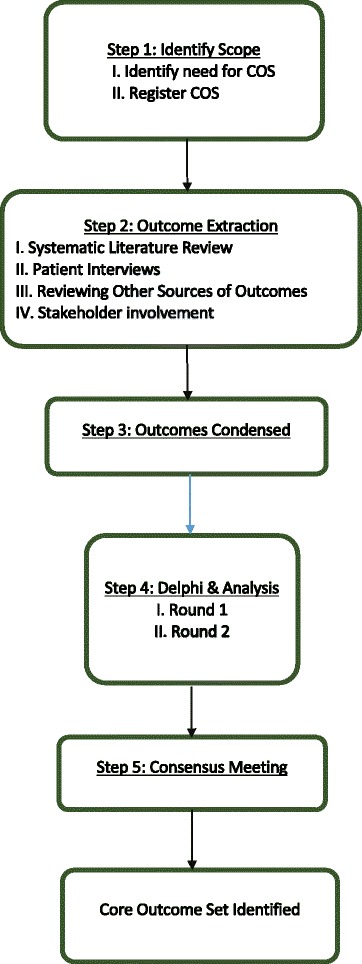
Flowchart of study design *COS* Core outcome set

We will adhere to the recommendations of both the COMET and CSG-COUSIN initiatives, with reporting conforming to the SPIRIT (Standard Protocol Items: Recommendations for Interventional Trials) checklist (Additional file 1). A brief overview of our study design is presented in Fig. 1 depicting a prior study protocol (adapted from [14]).

### Scope

This COS is intended as the global/international standard for clinical trials evaluating treatments for the physical appearance of facial aging. The COS to be developed may be applied to individuals of all ages, genders, skin types, races, and ethnicities.

### Identification of outcomes

Outcomes will be generated over four phases.

#### Phase I: systematic literature review

Randomized controlled trials on the physical appearance of facial aging, including both chronological aging and photoaging, will be reviewed with extraction of their reported outcomes.

#### Phase II: patient interviews

Patient interviews will be conducted to determine which patient-centered outcomes should be assessed.

#### Phase III: reviewing other sources of outcomes

Clinical trial registries and educational and treatment brochures will be reviewed to add to the long list any outcomes as yet uncovered.

#### Phase IV: stakeholder involvement

Nonpatient stakeholders will provide insight regarding additional outcomes they would like to see included in the COS.

### Literature review

A systematic literature review will be conducted using search terms related to facial aging. The query will be completed using PubMed, MEDLINE, Embase, CENTRAL, CINAHL, and the Cochrane Library and clinical databases. Included studies will be composed of randomized controlled trials related to facial aging. Article titles will be reviewed by four Measurement of Priority Outcome Variables in Dermatologic Surgery (IMPROVED) committee members and either approved or rejected through mutual agreement. Duplicate studies will be removed and included only once. Afterward, abstracts will be reviewed by the same four members for inclusion or exclusion. The remaining articles will then be extracted for data using a standard data extraction table. Authors, years of publication, sources of funding, and treatment comparisons will be documented. Length of follow-up, treatment period, results, outcomes, and outcome measures will be noted along with the indication for treatment (e.g., wrinkles, volume loss).

Outcome extraction will be performed with the help of nine data extractors across four institutions. The institutions involved will include Northwestern University Feinberg School of Medicine, St. Louis University School of Medicine, Penn State Hershey Medical Center, and the University of Pennsylvania Perelman Center for Advanced Medicine. Outcomes will then be consolidated by two to four IMPROVED investigators. Similar outcomes will be combined and listed only once without loss of content.

### Patient-centered outcomes

A semistructured interview will be conducted to explore other potential patient-identified outcomes. Interviews will be conducted with approximately 10–15 patients concerned with facial aging. A global context will be provided by including patients both in the United States and internationally. Interviews will be composed primarily of open-ended questions to elicit patient thoughts and opinions. Each interview will be recorded and transcribed for documentation of possible outcomes obtained during the interview. Through the use of interviews, we hope to provide a more complete account of issues important to patients.

### Additional sources

Outcomes from clinical trials registries, Cochrane reviews, and patient pamphlets and brochures will be extracted and included in the final list as well.

### Stakeholder involvement

As part of phase IV, stakeholder involvement will be elicited. Stakeholders are defined as those invested in the development of a COS in facial aging. Table 1, adapted from a prior protocol, lists potential stakeholders, which include dermatologists, plastic surgeons, drug and device safety regulators (e.g., U.S. Food and Drug Administration, European Medicines Agency), pharmacologists, pharmacists, industry scientists, nurses, physician assistants, and other health care providers [14].

**Table 1:** Summary of stakeholder involvement

Stakeholders
Physicians (including dermatologists, plastic surgeons, international providers, physicians of other health care fields)
Patients
Nurses, physician assistants, or other health care providers
Industry scientists
Cosmetic groups/support groups
Pharmacologists/pharmacists
Drug and device safety regulators (e.g., FDA, EMA)

*EMA* European Medicines Agency, *FDA* U.S. Food and Drug Administration

### Potential outcomes

The long list of outcomes obtained from the steps described above will then be examined by the steering committee, composed of four dermatologists: MA (Northwestern University), IAM (Saint Louis University), JFS (University of Pennsylvania), and TVC (Pennsylvania State University). Members may add or remove outcomes prior to the Delphi process. The steering committee members will not join in the Delphi process but will be invited to participate in the final consensus meeting.

### Delphi overview

Delphi surveys have been used in prior COS research [15]. The process involves using online surveys to collect opinions from participants on a particular topic through a series of rounds of data collection and analysis to condense the opinions of individuals into a group consensus. Responses will be analyzed and outcomes added or removed on the basis of participant input from each round. We plan on conducting two Delphi rounds prior to the consensus meeting.

### Participants

Two homogeneous groups made up of patients or physicians, respectively, will participate in the Delphi exercises. Groups will consist of approximately 30 individuals to allow for greater diversity of opinions and account for potential dropouts. Patients and physicians will be recruited both in the United States and internationally to provide a global context. Prior to the exercise, the Delphi process will be explained and demographic/occupational information obtained, including years of experience, field of interest, and position. Completion of the survey will imply consent to continue with the Delphi process. Participants will have 3 weeks to complete the online survey with email reminders sent at the 1- and 2-week marks. For each round, the number of participants invited and those who completed the surveys will be documented.

### Delphi rounds

In the first Delphi round, the complete list of outcomes gathered will be presented for rating. Outcomes will be listed randomly after each round to avoid any influence the order may have on participants. Scoring for each outcome will be performed using the scale devised by the Grading of Recommendations Assessment, Development and Evaluation (GRADE) working group. In this scale, participants can rate outcomes numerically on a scale of 1–9 (7–9 being critical, 4–6 being important, and 1–3 being of limited importance) [16]. The first round will also include a score of 10 to signify uncertainty if the outcome merits inclusion in the set. As discussed by the GRADE working group, this scale will allow participants to focus on ranking the most valued outcome high and exclude outcomes of lesser importance. All outcomes will be carried to the next round.

Descriptive statistics will be used to analyze the data from the two groups. Responses from both the patient and physician groups will be summarized and fed back. Participants will then be given the opportunity to use this information to alter the scoring of outcomes. New outcomes will be added only if suggested by two or more participants, with any uncertainties being addressed by the steering committee.

The second Delphi round will follow the same format as the previous round. The set of outcomes resulting from this second round will be presented at the consensus meeting.

### Consensus meeting

At the consensus meeting, the group will be presented with results from the final Delphi round. Outcomes will be retained and removed from the final list on the basis of the following terms of consensus: If 70% of participants rank an outcome 7–9 with less than 15% ranking it 1–3, it will be retained; if 70% of participants rank an outcome 1–3 and less than 15% rank it 7–9, it will be removed [17].

Discussion of each outcome will then be held with the help of a trained moderator. Items will anonymously be voted yes or no for inclusion in the final COS using live polling software. The end result will be a COS that can be agreed upon by patients, physicians, and other stakeholders.

### Core outcome measures

Facial aging is an ill-defined term that precludes measurement by a single metric. Our goal is to determine outcome measures to represent both biological aging and photoaging. For example, the Fitzpatrick wrinkle scale, though imperfect, is widely used and sufficient to complete and develop a COS. Once a COS has been developed, the HOME roadmap will be used for developing a core set of measures to assess the outcomes selected [13]. A systematic review covering at least two databases will be performed to identify current instruments and outcome measures used in clinical trials.

Using the COnsensus-based Standards for the selection of health measurement INstruments (COSMIN) framework for guidance, quality of the studies will be assessed by rating their validity, reliability, responsiveness to change, and interpretability. The COSMIN checklist provides a standard 4-point rating for each of these metrics. Reliability encompasses internal consistency, reliability, and measurement error. Validity encompasses content validity, construct validity, and criterion validity. Responsiveness to change and interpretability do not encompass additional subtopics.

To determine which measurements are suitable per outcome domain, a consensus meeting with key stakeholders, patients, and clinicians will be held [13]. Results from the systematic review will be provided to guide discussion. Members will then judge the measures on the basis of how valid, reliable, and feasible they may be for assessing each core outcome domain. New instruments will be developed if there is inadequate evidence supporting existing methods. At the end of the consensus meeting, relevant stakeholders will vote and recommend an outcome measurement instrument per core outcome domain.

## Discussion

COSs have been proposed as a solution to selective outcome reporting. There is currently no COS relevant to clinical trials of facial aging. The proposed study will incorporate the opinions of patients, physicians, and other key stakeholders to create such a set to eliminate the inconsistency of outcomes and outcome measurements examined across relevant trials. Through the use of COSs, we hope to improve research trials and clinical practice.

### Trial status

The development of the COS is active and ongoing in its initial phase of outcome extraction.

## Additional file

Additional file 1: SPIRIT checklist. Completed checklist of the study protocol for the development of a core outcome set. (DOCX 52 kb)

## Abbreviations

COMET: Core Outcome Measures in Effectiveness Trials
COS: Core outcome set
COSMIN: COnsensus-based Standards for the selection of health measurement Instruments
GRADE: Grading of Recommendations Assessment, Development and Evaluation
CSG-COUSIN: Cochrane Skin Group - Core Outcome Set Initiative
EMA: European Medicines Agency
FDA: U.S. Food and Drug Administration
HOME: Harmonising Outcome Measures for Eczema
IMPROVED: Measurement of Priority Outcome Variables in Dermatologic Surgery
SPIRIT: Standard Protocol Items: Recommendations for Interventional Trials
